# Noninvasive Targeted Crohn Disease Management by Combining Endoscopic Healing Index and Therapeutic Drug Monitoring

**DOI:** 10.1093/crocol/otab035

**Published:** 2021-06-09

**Authors:** Amy Hemperly, Marla C Dubinsky, Andres Yarur, Anita Afzali, Stephen Hanauer, Subra Kugathasan, Millie D Long, Shervin Rabizadeh, Robbyn Sockolow, Lauren Okada, Anjali Jain, Maria T Abreu, Niels Vande Casteele

**Affiliations:** 1 Department of Pediatrics, Division of Gastroenterology, University of California San Diego, La Jolla, California, USA; 2 Rady Children’s Hospital, San Diego, California, USA; 3 Division of Pediatric Gastroenterology and Nutrition, Icahn School of Medicine, New York, New York, USA; 4 Division of Gastroenterology and Hepatology, Medical College of Wisconsin, Milwaukee, Wisconsin, USA; 5 Division of Gastroenterology, Hepatology, and Nutrition, The Ohio State University Wexner Medical Center, Columbus, Ohio, USA; 6 Division of Gastroenterology and Hepatology, Northwestern University-Feinberg School of Medicine, Chicago, Illinois, USA; 7 Division of Pediatric Gastroenterology, Emory University School of Medicine, Atlanta, Georgia, USA; 8 Department of Medicine, Division of Gastroenterology and Hepatology, University of North Carolina at Chapel Hill, Chapel Hill, North Carolina, USA; 9 Department of Pediatrics, Cedars-Sinai Medical Center, Los Angeles, California, USA; 10 Department of Pediatrics, Division of Gastroenterology and Nutrition, Weill Cornell Medicine, New York, New York, USA; 11 Prometheus Biosciences, San Diego, California, USA; 12 Department of Medicine, Division of Gastroenterology, Crohn’s & Colitis Center, University of Miami Miller School of Medicine, Miami, Florida, USA; 13 Department of Medicine, University of California San Diego, La Jolla, California, USA

**Keywords:** inflammatory bowel disease, endoscopic remission, treat-to-target, biomarker

## Abstract

**Background and Aims:**

Therapeutic drug monitoring (TDM) with measurement of serum drug and antidrug antibody concentrations is used to optimize tumor necrosis factor antagonists (anti-TNF). The endoscopic healing index (EHI) is a validated serum-based assay to measure mucosal inflammation in adults with Crohn disease (CD). Our objectives were to evaluate the relationship between EHI and TDM results and to determine the anti-TNF concentration range associated with EHI <20 (consistent with endoscopic remission).

**Methods:**

Adult and pediatric patients with CD (N = 1731) were selected retrospectively from a clinical laboratory cohort. Patients were selected if they had an ICD-10 code for CD and if results for EHI and TDM were available within 30 days of each other. The relationship between EHI and TDM results was examined and the anti-TNF concentration range associated with EHI <20 vs >50 was evaluated.

**Results:**

Median anti-TNF concentration was higher in patients with EHI <20 vs >50 for infliximab (N = 796): 11.1 vs 3.4 µg/mL and for adalimumab (N = 935): 9.2 vs 5.0 µg/mL (*P* < 0.0001 both drugs). Patients with antibodies to infliximab (12.8%) or adalimumab (14.9%) had lower anti-TNF concentrations (*P* < 0.001 both drugs) and higher EHI (*P* < 0.01 both drugs). The concentration range for infliximab: 5–15 µg/mL (5–9 µg/mL in pediatric patients) and for adalimumab: 5–10 µg/mL (8 µg/mL in pediatric patients) best discriminated EHI <20 vs >50.

**Conclusions:**

We report the anti-TNF concentration range associated with EHI <20. Combined testing of EHI and TDM is proposed as a noninvasive approach for treat-to-target management which could improve the ability to monitor disease and optimize anti-TNF therapy.

## Introduction

Crohn disease (CD) is a chronic and progressive immune-mediated inflammatory disorder with substantial morbidity.^1^ Early initiation of antitumor necrosis factor (TNF) therapy can prevent disease-related complications in patients with CD identified to be at high risk for disease progression.^2, 3^ Furthermore, utilizing both a symptom-based and biomarker-based monitoring strategy to inform timely treatment decisions (ie, treat-to-target) can result in better clinical and endoscopic outcomes.^4^ The recommended therapeutic target in CD is both clinical and endoscopic remission.^5^ Achievement of endoscopic remission is associated with improvements in quality of life, sustained steroid-free remission, and fewer CD-related hospitalizations.^6, 7^ However, relying on endoscopic assessment to ascertain endoscopic remission as a therapeutic endpoint carries a high burden for patients.^5, 8^ This is particularly true for pediatric patients because repeat endoscopic assessments under general anesthesia and scoring of endoscopic activity are not commonly performed.

The use of noninvasive surrogate markers of endoscopic remission may improve disease monitoring in “real-world” practice. C-reactive protein and the Crohn Disease Activity Index are unreliable markers of endoscopic disease activity. Fecal calprotectin is a reliable noninvasive surrogate marker of endoscopic activity and remission but limitations of using fecal calprotectin as a biomarker for endoscopic disease activity include difficulty in sample collection and variability related to platform, disease location, phenotype, collection technique, and timing of sample collection.^9–11^ The endoscopic healing index (EHI) is a novel, validated serum-based assay for mucosal inflammation in CD with reproducibility and favorable consistency in performance across disease locations and phenotypes.^12^ The EHI score ranges from 0 to 100 units with higher scores indicating more severe endoscopic disease activity. An EHI <20 was observed to have high sensitivity for ruling out endoscopic inflammation (simple endoscopic score for CD of <2 and for each segment <1, or a total CD endoscopic index of severity score <3), and an EHI >50 was observed to have a high specificity for ruling in endoscopic inflammation (simple endoscopic score for CD >2 or simple endoscopic score for CD = 2 if only 1 segment had a score of 2 with a score of 0 in the remaining segments).

The composite outcome of clinical and endoscopic remission in CD can be achieved with anti-TNF therapy.^13–16^ Higher infliximab and adalimumab serum drug concentrations have been associated with achieving these stringent outcomes, and thus therapeutic drug monitoring (TDM) has been proposed to optimize therapy.^7, 17–26^ In the current study, we evaluated the correlation between TNF antagonist serum concentrations and the EHI, and determined the TNF antagonist concentration range associated with an EHI <20, which is consistent with endoscopic remission. We also propose a noninvasive treat-to-target approach that incorporates both the EHI and TDM, which could improve our ability to monitor and improve the therapeutic efficacy of TNF antagonists.

## Methods

Adult and pediatric patients were selected retrospectively from a cohort of samples submitted for analysis to a commercial clinical laboratory (Prometheus Biosciences, San Diego, CA, USA). Pediatric patients were defined as persons aged 18 years or younger. De-identified information was extracted from the clinical laboratory database. Clinical information that did not include the Health Insurance Portability and Accountability Act (HIPAA) identifiers was used for analyses. Data extraction was reviewed and approved by the Prometheus Biosciences privacy office for compliance. Patients were selected if they had an ICD-10 code for CD and EHI and anti-TNF concentration results were available within 30 days of each other. The EHI, anti-TNF concentrations, and antidrug antibody concentrations were determined as previously published.^12, 27^ The relationship between the EHI, anti-TNF concentrations, and antidrug antibody concentrations was examined.

### Statistical Analysis

Data analysis included descriptive statistics computed for continuous variables (mean and SD for normally distributed data, median with interquartile rage [IQR] for non-normally distributed data), and percentages for categorical variables. Continuous variables were compared between 2 groups using the Mann–Whitney *U* test and Dunn test with Bonferroni correction for multiple comparisons. χ ^2^ test of independence was used for calculating *P* values between EHI binary groups and drug quartiles for both infliximab and adalimumab except for the adalimumab pediatric population where a Fisher exact test was used due to a small sample size. A sensitivity analysis was performed to determine the association of high-titer (>10 U/mL) antidrug antibodies with an EHI <20 and an EHI >50. Univariate and multivariate analyses were conducted to explore variables associated with an EHI <20. Receiver operating characteristic (ROC) curves were evaluated to identify anti-TNF concentration thresholds associated with an EHI <20 with 80% specificity and an EHI >50 with 80% sensitivity, instead of the Youden index which provides the best tradeoff between sensitivity and specificity. ROC analysis did not include samples with an EHI between 20 and 50. Thresholds were rounded to the nearest whole number. An α level of 5% was considered as threshold for significance. All data analysis was carried out using R version 3.6.2.

## Results

### Demographics and Disease Characteristics

The study included results from 1731 samples from unique patients (1444 adult and 287 pediatric patients) reported before August 31, 2019. EHI and anti-TNF concentrations were available from the same sample in 88% of patients. Demographic data are shown in Table 1. The mean age for patients treated with infliximab (N = 796) was 36 years; SD, 20 years; 26% <18 years of age. The mean age for patients treated with adalimumab (N = 935) was 43 years; SD, 18 years; 9% <18 years of age. Gender was balanced within anti-TNF groups (47% of patients treated with infliximab and 46% of patients treated with adalimumab were male).

**Table 1.** Patient Demographics

	Anser IFX	Anser ADA
# of Patients	796	935
Males, %	47%	46%
Age, entire cohort [Mean (SD)], years	35.9 (20.0)	42.5 (18.4)
Patients ≤18 years, %	25.8%	8.8%
Patients >18 years, %	74.2%	91.2%
Patients with complete biologic dosing info, N (%)	282 (35.4%)	488 (52.2%)
EHI, N (%)		
EHI <20	245 (30.8%)	246 (26.3%)
EHI ≤20 vs ≤50	405 (50.9%)	488 (52.2%)
EHI >50	146 (18.3%)	201 (21.5%)

Anser ADA, Prometheus serum adalimumab level; Anser IFX, Prometheus serum infliximab level.

The median [interquartile range, IQR] infliximab concentration was 9.35 µg/mL [3.40–19.18] and the median adalimumab concentration was 8.0 µg/mL [4.1–12.1]. Infliximab concentrations were not significantly different between adult and pediatric patients whereas adalimumab concentrations were noted to be higher in pediatric patients than adult patients (*P* = 0.0121) (Supplementary Table 1). Using the suggested target trough concentrations during maintenance therapy of 5 µg/mL for infliximab and 7.5 µg/mL for adalimumab,^28^ 33% of patients treated with infliximab, and 47% of patients treated with adalimumab had subtherapeutic concentrations.

The median EHI was 28 [17–45] in patients treated with infliximab and 32 [19–47] in patients treated with adalimumab. The EHI was <20 in 31% and >50 in 18% of patients treated with infliximab whereas the EHI was <20 in 26% and >50 in 22% of patients treated with adalimumab. The EHI was significantly lower in pediatric patients than in adult patients receiving infliximab or adalimumab (*P* < 0.0001 and *P* = 0.0006, respectively) (Supplementary Table 1). Approximately 55% of pediatric patients receiving infliximab and 45% of pediatric patients receiving adalimumab had an EHI <20 whereas 20% of adult patients receiving infliximab and 25% of adult patients receiving adalimumab had an EHI <20.

### Antidrug Antibodies

Anti-infliximab antibodies were detected in 102/796 patients (12.8%), and anti-adalimumab antibodies were detected in 139/935 patients (14.9%). There was a significant inverse correlation between serum drug concentrations and antidrug antibodies on univariate analysis. Patients with anti-infliximab antibodies compared to those without anti-infliximab antibodies had a lower median infliximab concentration (<1.0 µg/mL vs 10.6 µg/mL; *P* < 0.001) and higher median EHI (38 vs 28; *P* = 0.001). Patients with anti-adalimumab antibodies compared to those without anti-adalimumab antibodies had a lower median adalimumab concentration (<1.6 µg/mL vs 9 µg/mL; *P* < 0.001) and higher median EHI (38 vs 31; *P* = 0.008) (Supplementary Fig. 1). High-titer (>10 U/mL) anti-infliximab antibodies were detected in 56/102 patients (54.9%) and high-titer (>10 U/mL) anti-adalimumab antibodies were detected in 32/139 patients (23.0%). Patients with high-titer antidrug antibodies were more likely to have an EHI >50 than an EHI <20 (infliximab 25% vs 13%; adalimumab 41% vs 16%). Therefore, the lowest percentage of high-titer antibodies were observed in patients with EHI <20.

### Exposure–Response Relationship

**Figure 1.**
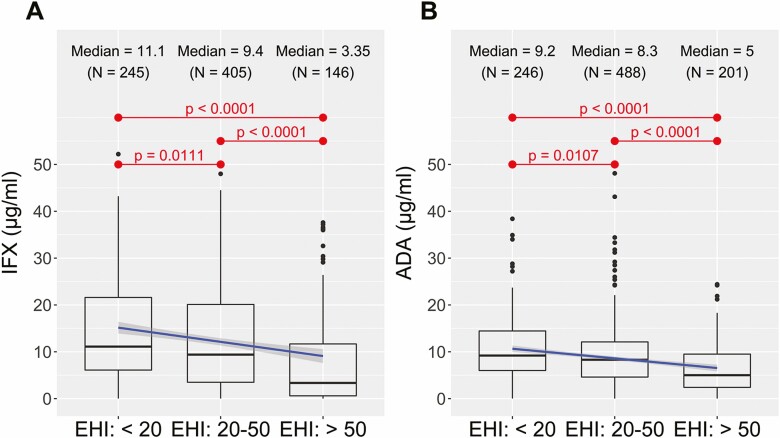
Median serum drug concentration of infliximab (A) and adalimumab (B) per EHI category of <20, between 20 and 50, and >50.

**Figure 2.**
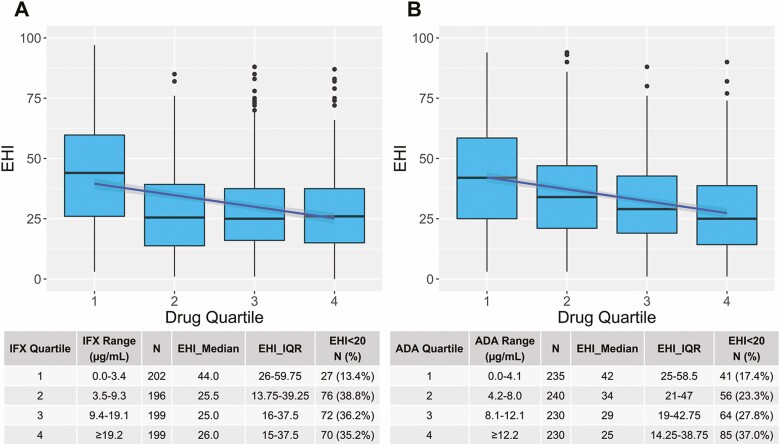
Linear exposure–response relationship between median EHI and serum drug concentration quartiles in all patients with CD treated with infliximab (A) or adalimumab (B). ADA, adalimumab; IFX, infliximab.

**Figure 3.**
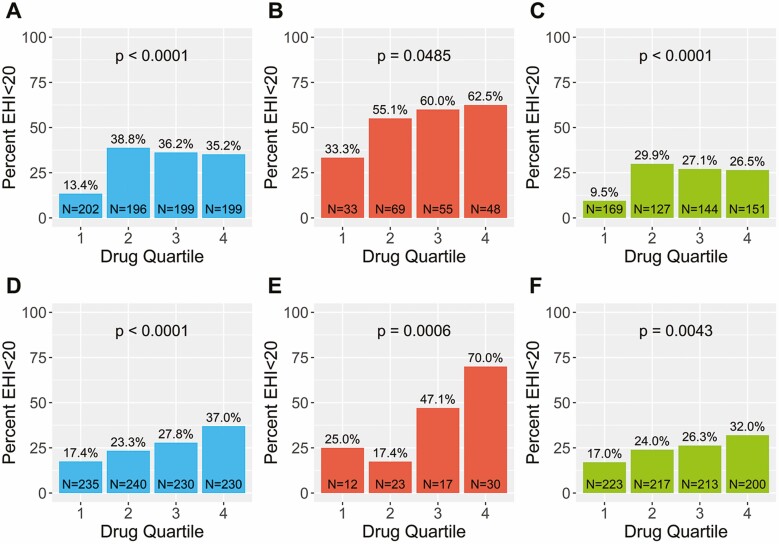
Proportion of CD patients with an EHI <20 by serum concentration quartile of infliximab in the overall population (A), pediatric subpopulation (B) and adult subpopulation (C); or adalimumab in the overall population (D), pediatric subpopulation (E), and adult subpopulation (F). Quartiles for infliximab: Q1, ≤3.4 µg/mL; Q2, 3.5–9.3 µg/mL; Q3 9.4–19.1 µg/mL, Q4 ≥ 19.2 µg/mL. Quartiles for adalimumab: Q1, ≤4.1 µg/mL; Q2, 4.2–8.0 µg/mL; Q3 8.1–12.1 µg/mL, Q4 ≥12.2 µg/mL.

There was a significant inverse correlation between serum infliximab and adalimumab concentrations and EHI in pediatric and adult patients on both univariate and multivariate analysis. For the overall cohort, the median infliximab concentration was higher for an EHI <20 compared to an EHI >50 (11.1 µg/mL [IQR 6.1–21.6] vs 3.4 µg/mL [IQR 0.6–11.7], *P* < 0.0001) and the median adalimumab concentration was higher for an EHI <20 compared to an EHI >50 (9.2 µg/mL [IQR 6.0–14.4] vs 5.0 µg/mL [IQR 2.4–9.5], *P* < 0.0001) (Fig. 1). Similar results were obtained for pediatric and adult patients separately (Supplementary Fig. 2). A significant linear inverse exposure–response relationship was observed between the median EHI across infliximab and adalimumab concentration quartiles in the overall cohort (*P* < 0.001 both drugs) (Fig. 2) and for adult and pediatric patients separately (Supplementary Figs. 3 and 4). Vice versa, for both infliximab and adalimumab, a significant linear exposure–response relationship was observed whereby higher quartiles of drug concentrations were associated with a higher proportion of patients achieving an EHI <20 (Fig. 3).

### Threshold Analysis

**Figure 4.**
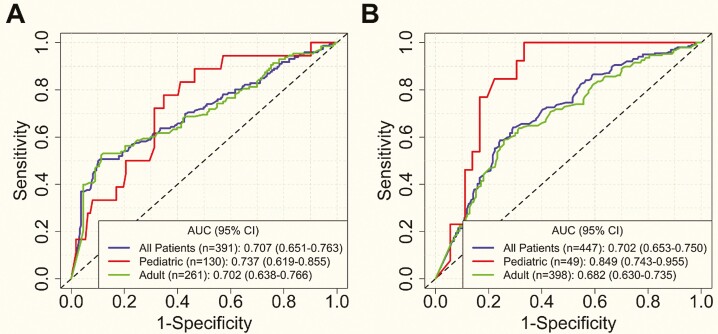
ROC curves of serum drug concentrations discriminating EHI of <20 vs >50 for infliximab (A) and adalimumab (B). CI, confidence interval.

Serum drug concentration thresholds associated with an EHI <20 and an EHI >50 were evaluated using ROC curve analysis (Fig. 4). An infliximab therapeutic range of 5–15 µg/mL (5–9 µg/mL in pediatric patients) was found where a concentration of ≥5 µg/mL had a specificity of ≥80% to detect subjects with an EHI <20 and a concentration of <15 µg/mL (<9 µg/mL in pediatric patients) had a sensitivity of ≥80% to detect subjects with an EHI >50 (Table 2). An adalimumab therapeutic range of 5–10 µg/mL (8 µg/mL in pediatric patients) was found where a concentration of ≥5 µg/mL (≥8 µg/mL in pediatric patients) had a specificity of ≥80% to detect subjects with an EHI <20 and a concentration of <10 µg/mL (<8 µg/mL in pediatric patients) had a sensitivity of ≥80% to detect subjects with an EHI >50 (Table 2).

**Table 2.** Receiver Operating Characteristic (ROC) Curve Analysis for Drug Concentrations Distinguishing Endoscopic Healing Index (EHI) >50 vs <20

Infliximab	Threshold, µg/mL	AUROC (95% CI)	Sensitivity (95% CI)	Specificity (95% CI)	PLR (95% CI)	NLR (95% CI)
*Drug cutoffs for differentiating EHI >50 vs EHI <20 with 80% specificity*						
Overall, N = 391	5.0	0.707 (0.651–0.763)	54.1 (45.7–62.4)	80.0 (74.4–84.8)	2.705 (2.021–3.621)	0.574 (0.476–0.692)
Adult, N = 261	5.0	0.702 (0.38–0.766)	56.2 (47.2–65.0)	79.7 (71.9–86.2)	2.771 (1.914–4.011)	0.549 (0.443–0.680)
Pediatric, N = 130	5.1	0.737 (0.619–0.855)	38.9 (17.3–64.3)	80.4 (71.8–87.3)	1.980 (0.993–3.946)	0.760 (0.520–1.112)
*Drug cutoffs for differentiating EHI >50 vs EHI <20 with 80% sensitivity*						
Overall, N = 391	14.5	0.707 (0.651–0.763)	80.1 (72.7–86.3)	38.4 (32.2–44.8)	1.300 (1.144–1.477)	0.518 (0.360–0.744)
Adult, N = 261	15.3	0.702 (0.38–0.766)	79.7 (71.7–86.3)	36.8 (28.6–45.6)	1.262 1.079–1.475)	0.551 (0.366–0.830)
Pediatric, N = 130	8.7	0.737 (0.619–0.855)	83.3 (58.6–96.4)	58.9 (49.2–68.1)	2.029 1.498–2.747)	0.283 (0.100–0.804)
Adalimumab	Threshold, µg/mL	AUROC (95% CI)	Sensitivity (95% CI)	Specificity (95% CI)	PLR (95% CI)	NLR (95% CI)
*Drug cutoffs for differentiating EHI >50 vs EHI <20 with 80% specificity*						
Overall, N = 447	4.6	0.702 (0.653–0.750)	45.3 (38.3–52.4)	80.9 (75.4–85.6)	2.370 (1.758–3.195)	0.677 (0.588–0.778)
Adult, N = 398	4.6	0.682 (0.630–0.735)	46.8 (39.5–54.2)	79.5 (73.4–84.8)	2.286 (1.682–3.108)	0.669 (0.575–0.778)
Pediatric, N = 49	8.0	0.849 (0.743–0.955)	76.9 (46.2–95.0)	80.6 (64.0–91.8)	3.956 (1.909–8.197)	0.286 (0.105–0.783)
*Drug cutoffs for differentiating EHI >50 vs EHI <20 with 80% sensitivity*						
Overall, N = 447	10.1	0.702 (0.653–0.750)	80.1 (73.9–85.4)	46.7 (40.4–53.2)	1.504 (1.313–1.723)	0.426 (0.313–0.579)
Adult, N = 398	10.1	0.682 (0.630–0.735)	79.8 (73.3–85.3)	42.4 (35.6–49.4)	1.385 (1.208–1.587)	0.477 (0.345–0.660)
Pediatric, N = 49	8.3	0.849 (0.743–0.955)	84.6 (54.6–98.1)	77.8 (60.8–89.9)	3.808 (1.981–7.320)	0.198 (0.055–0.716)

EHI >50 was considered as the positive or active disease and EHI <20 was considered as the negative or inactive disease. See Fig. 4 and Supplementary Tables 2 and 3 for additional details.

AUROC, area under the receiver operating characteristic curve; CI, confidence interval; PLR, positive likelihood ratio; NLR, negative likelihood ratio.

In the overall cohort, when retrospectively applying thresholds, 146/391 (37.3%) patients treated with infliximab were identified with an EHI >50 of whom 117/146 (80.1%) had an infliximab concentration <15 µg/mL (Supplementary Table 2). Similarly, 201/447 (45.0%) patients treated with adalimumab were identified with an EHI >50 of whom 161/201 (80.1%) had an adalimumab concentration <10 µg/mL (Supplementary Table 3). Approximately 20% of patients on infliximab and adalimumab were identified to have mechanistic failure (EHI >50 with infliximab concentration >15 µg/mL or adalimumab concentration >10 µg/mL, respectively).

## Discussion

In previous studies, the recommended threshold trough concentrations for TNF antagonists when using reactive TDM during maintenance therapy (infliximab ≥5 µg/mL and adalimumab ≥7.5 µg/mL) were derived largely from cross-sectional studies using clinical disease activity measures to initiate an intervention and to measure success of an intervention.^29^ However, assessment of disease activity and response to therapy has moved beyond symptom-based measures to mucosal healing. Currently, there is limited knowledge about the optimal threshold concentrations associated with mucosal healing, particularly for CD.^30^ In this study, we investigated the association between serum TNF antagonist concentrations and the EHI, a noninvasive biomarker with favorable diagnostic accuracy for identifying mucosal inflammation for patients with CD. An inverse exposure–response relationship was observed between serum anti-TNF concentrations and the EHI with lower anti-TNF serum concentrations being associated with a higher EHI. When assessed by anti-TNF concentration quartiles, patients in higher anti-TNF concentration quartiles were significantly more likely to have an EHI <20 compared to those in lower anti-TNF concentration quartiles.

Additionally, we assessed the therapeutic range of anti-TNF concentrations based on a lower threshold with high specificity (≥80%) and an upper threshold with high sensitivity (≥80%) associated with an EHI <20 or >50, respectively. Similar to previously published thresholds to achieve mucosal healing,^19, 22, 23, 31, 32^ we observed the therapeutic range for infliximab was 5–15 µg/mL (5–9 µg/mL in pediatric patients) and the therapeutic range for adalimumab was 5–10 µg/mL (8 µg/mL in pediatric patients). We hypothesize that patients with serum infliximab concentration ≥15 µg/mL or adalimumab concentration ≥10 µg/mL with an EHI >50 may have reached a therapeutic ceiling and would unlikely benefit from further dose escalation, provided these measurements were timed close to the next infusion (ie, at trough). Our analyses show that patients with serum infliximab or adalimumab concentrations of <5 µg/mL had respectively a 3-fold or 2-fold higher likelihood of having an EHI >50 (positive likelihood ratio of 2.7 or 2.4). Our observations seem to indicate that higher serum adalimumab concentration thresholds should be targeted in pediatric vs adult patients with CD. Also, adalimumab, but not infliximab, concentrations were noted to be higher in pediatric patients than in adult patients. This is in line with a lower observed adalimumab clearance in pediatric compared to adult patients with CD (0.281 L/d vs 0.40 L/d) and a comparable infliximab clearance observed between pediatric and adult patients with CD.^33, 34^

**Figure 5.**
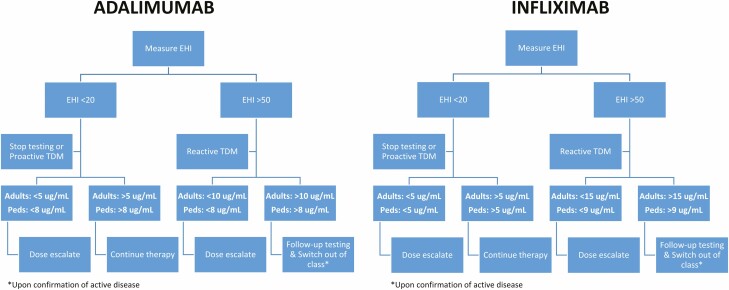
Proposed noninvasive approach for tight control management using EHI and anti-TNF concentrations for patients with CD.

In Fig. 5, we propose a noninvasive approach for tight disease control management using the EHI and serum anti-TNF concentrations in CD. Both EHI and anti-TNF concentrations can be measured in the same serum sample. The therapeutic target is achieved when the patient is within the therapeutic concentration range and the EHI is <20. However, in the setting of proactive TDM, dose escalation in patients with subtherapeutic anti-TNF concentrations may be considered. When the EHI is >50, there is high likelihood of active disease. In the setting of reactive TDM, dose escalation should be considered in patients with subtherapeutic anti-TNF concentrations. Follow-up testing (eg, endoscopy) and potentially switching to a treatment out of class may be considered in patients with therapeutic anti-TNF concentrations. The proposed treat-to-target algorithm, which incorporates TDM and EHI, serves as a conceptual framework for future studies. The algorithm and therapeutic thresholds remain to be validated in well-designed prospective studies with clinical data prior to use in practice. The optimal time and frequency of testing should also be studied.

This is the largest study to date to report target serum anti-TNF concentration thresholds associated with a serum-based biomarker for endoscopic disease activity and the first study to report target thresholds using the EHI for both adult and pediatric patients with CD. The strength of this study is that data were collected from a large, real-world, heterogeneous cohort of adult and pediatric patients with CD. The finding that over 1500 unique patient samples were available that had both anti-TNF levels and EHI measured simultaneously suggest that some clinicians are already utilizing this strategy for management of CD patients. Yet, it is important to acknowledge the limitations of the analysis. The main limitation was that data was extracted from a large laboratory database so clinical endpoints could not be included. The lack of patient-relevant clinical outcomes limits generalizability and did not allow us to study confounding factors that may affect the exposure–response analysis. Second, we were unable to control for nontrough samples or control for TDM performed reactively vs proactively in this retrospective cohort study. Last, the EHI remains to be validated in pediatrics. We noted a higher proportion of pediatrics patients compared to adults had an EHI <20. Potential explanations are that pediatric patients with CD may be more likely to experience endoscopic remission in response to TNF antagonists or that there may be differences in EHI cutoffs or in the weighting of the proteins measured in the EHI that remain to be adjusted for a growing population. We also do not know the reason for ordering TDM test (proactive vs reactive) and it may be likely that pediatric gastroenterologists are more proactive than reactive. In conclusion, here we describe the association between anti-TNF concentrations and the EHI and report on anti-TNF concentration thresholds associated with endoscopic remission (EHI <20). Combined testing of EHI and anti-TNF concentrations could identify patients who may benefit from dose escalation or who may have reached a therapeutic ceiling. TDM for other biologics and their relationship to EHI should additionally be explored.

## Supplementary Material

otab035_suppl_Supplementary_Materials

## Data Availability

Data not publicly available.
