# Supplementary material for: Noninvasive Targeted Crohn Disease Management by Combining Endoscopic Healing Index and Therapeutic Drug Monitoring
Source: Crohns Colitis 360. 2021 Jun 9;3(3):otab035. doi: 10.1093/crocol/otab035 (PMC9802228; doi:10.1093/crocol/otab035)
Supplement: otab035_suppl_Supplementary_Materials [file otab035_suppl_supplementary_materials.docx]

**SUPPLEMENTARY MATERIALS**

**SUPPLEMENTARY TABLES**

**Supplementary Table 1.** Serum drug concentration and endoscopic healing index per drug and patient population

|  | Infliximab | | | | Adalimumab | | | |
| --- | --- | --- | --- | --- | --- | --- | --- | --- |
|  | **Adults** | **Peds** | **All** | **p-value (Adults vs Peds)** | **Adults** | **Peds** | **All** | **p-value (Adults vs Peds)** |
| Drug concentration,  median [IQR] | 9.30  [2.85 – 19.75] | 9.90  [4.80 – 18.10] | 9.35  [3.40 – 19.18] | 0.2100 | 7.80  [3.90 – 11.90] | 8.70  [5.83-14.75] | 8.00  [4.10 – 12.10] | 0.0121 |
| EHI,  median [IQR] | 32  [20 – 49] | 18  [9 – 32] | 28  [17-45] | <0.0001 | 32  [20 – 48] | 24  [11 – 39. 75] | 32  [19 – 47] | 0.0006 |

**Supplementary Table 2.** Infliximab contingency tables based on a lower threshold with high specificity (≥80%) and an upper threshold with high sensitivity (≥80%) associated with an endoscopic healing index (EHI) of <20 or >50.

**
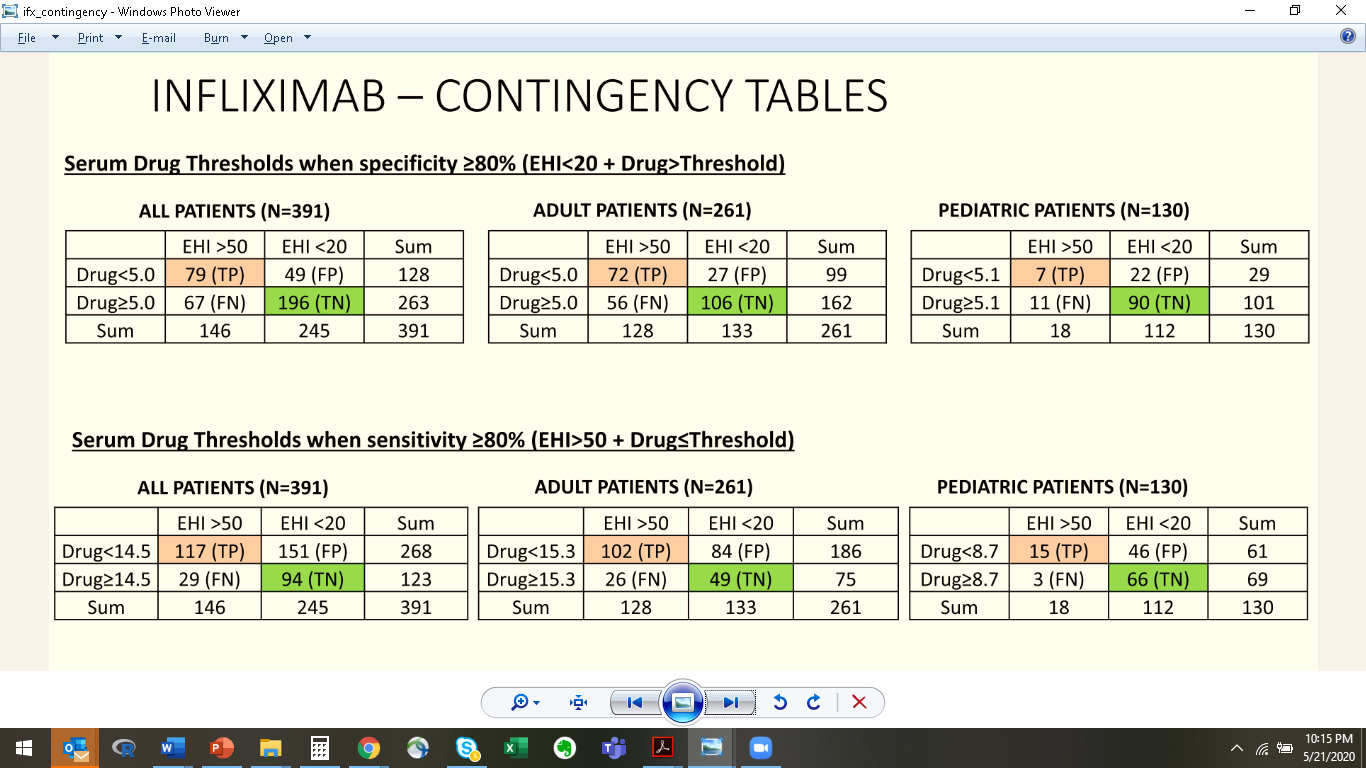
**

**Supplementary Table 3.** Adalimumab contingency tables based on a lower threshold with high specificity (≥80%) and an upper threshold with high sensitivity (≥80%) associated with an endoscopic healing index (EHI) of <20 or >50.

**
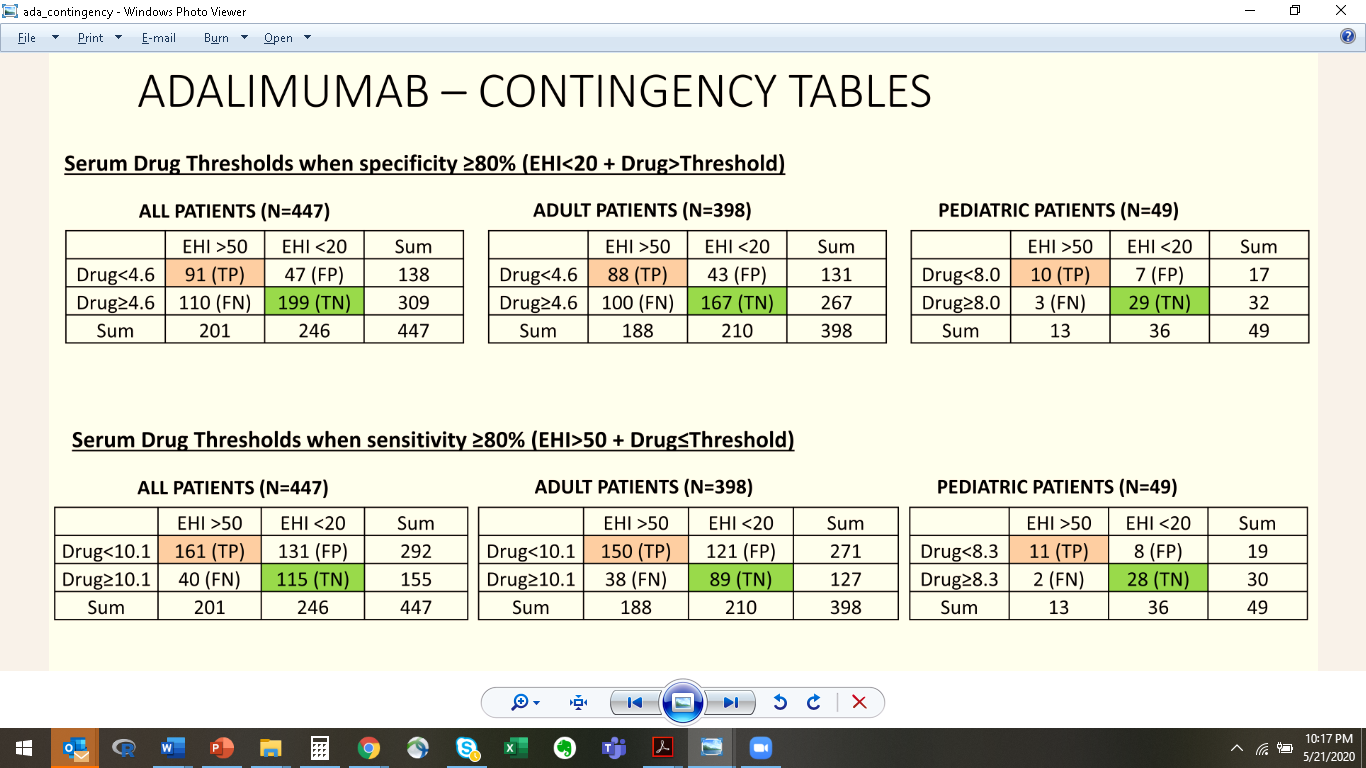
**

**SUPPLEMENTARY FIGURES**

**
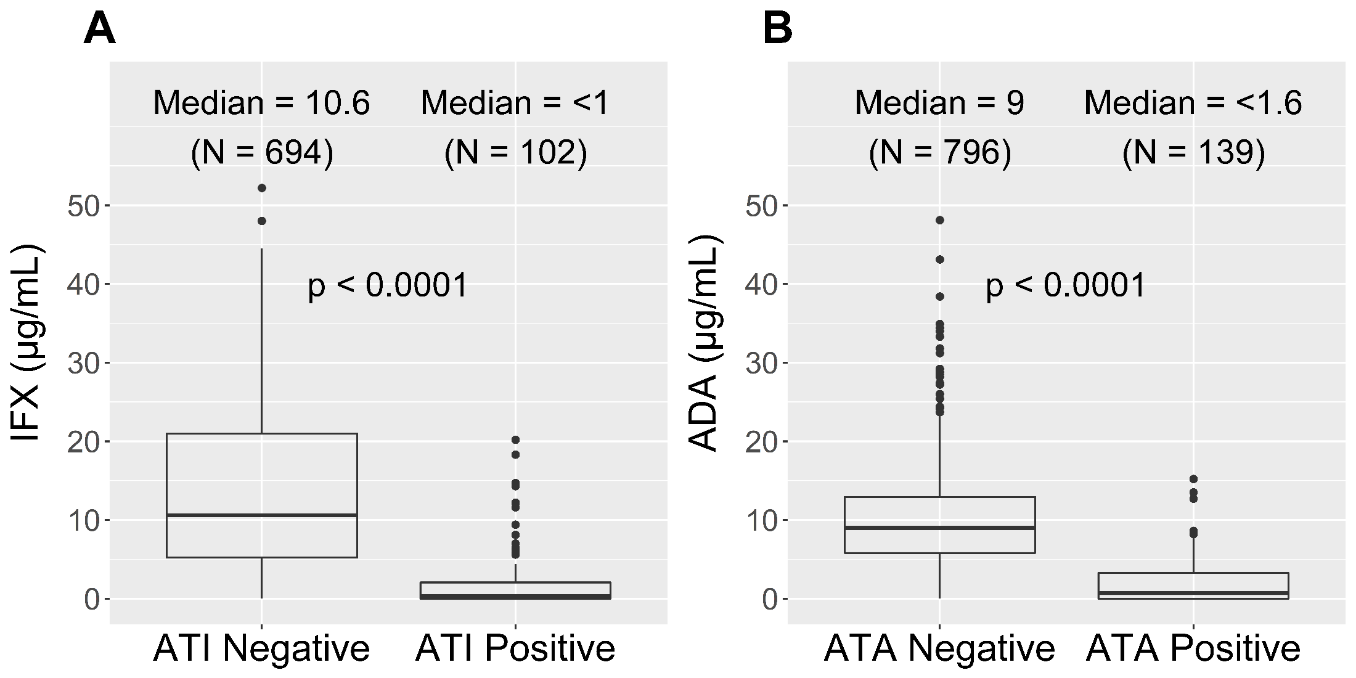

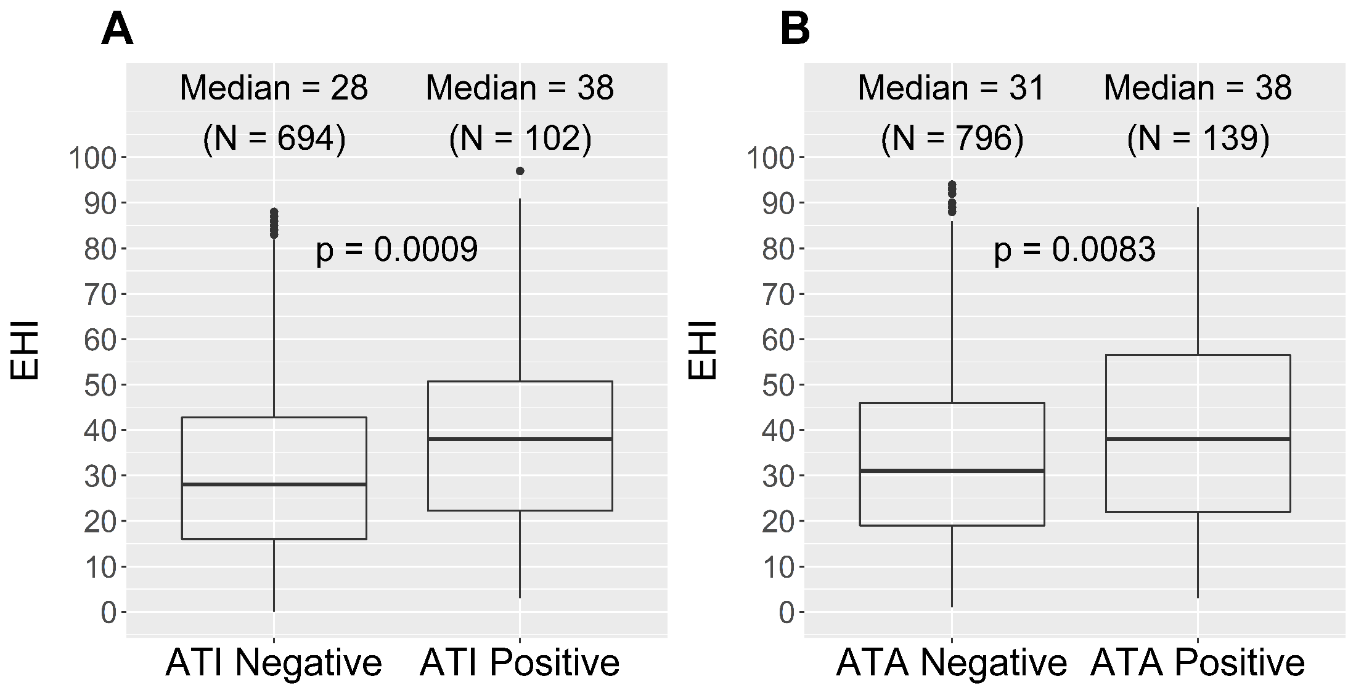
**

**Supplementary Figure 1.** Median serum drug concentrations (top panel) and median EHI scores (bottom panel) in antibody negative and antibody positive samples in infliximab (A) and adalimumab (B) treated patients.


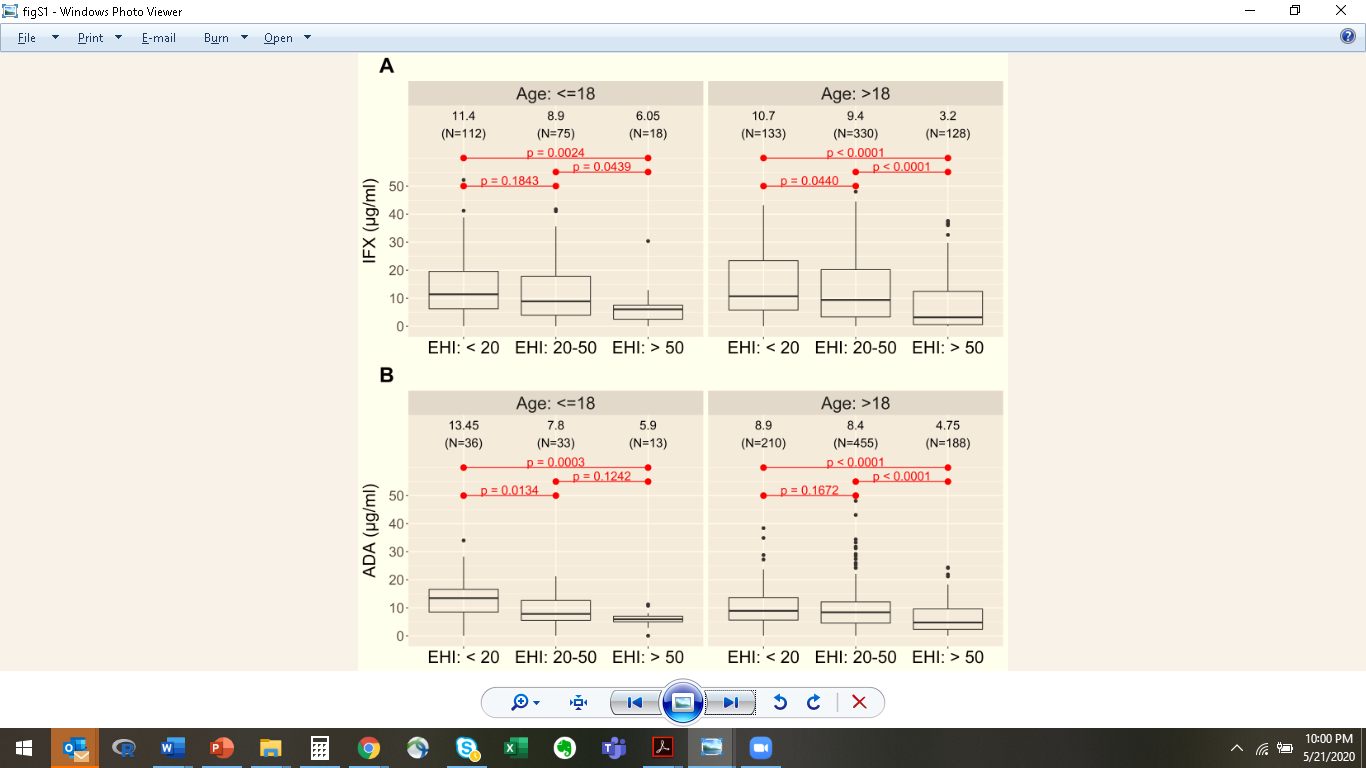


**Supplementary Figure 2.** Median serum drug concentration of infliximab (A) and adalimumab (B) per endoscopic healing index (EHI) category of <20,
between 20-50 and >50 faceted on age.


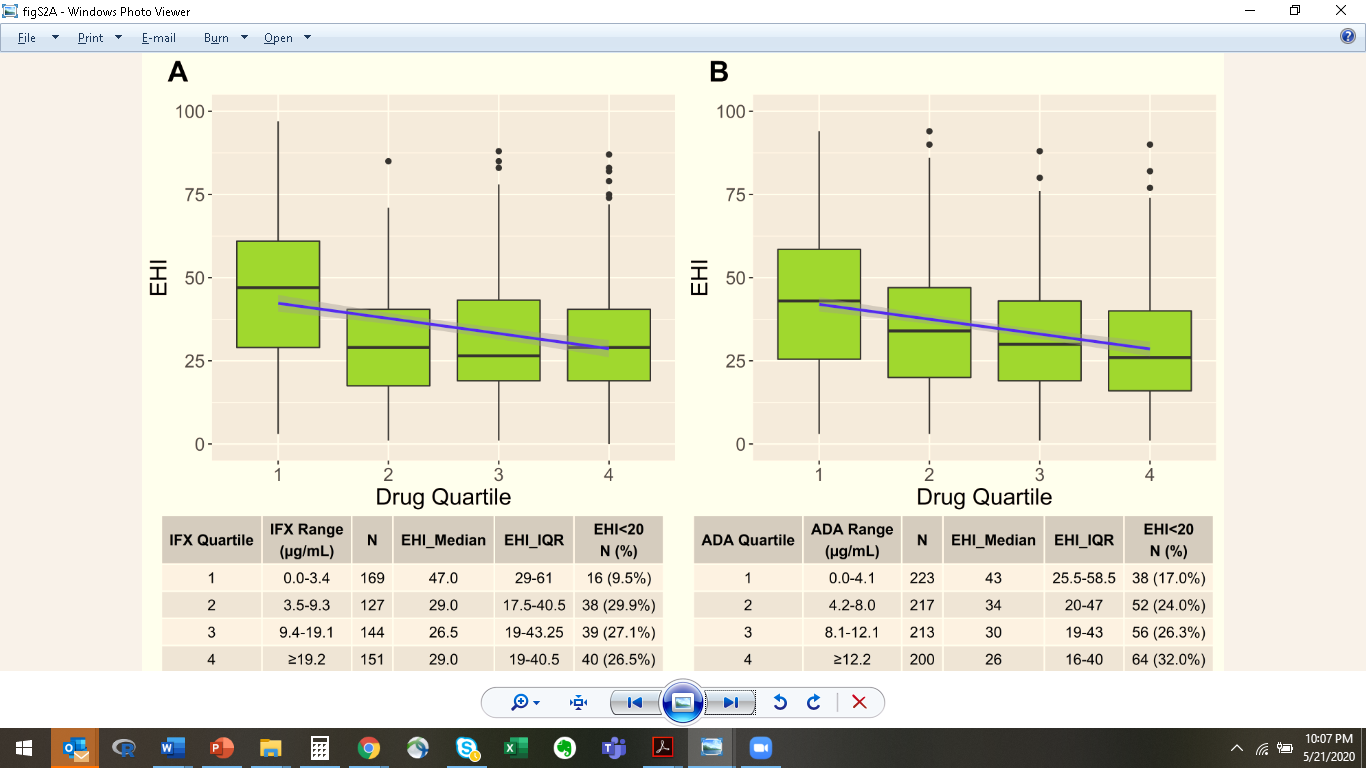


**Supplementary Figure 3.** Linear exposure-response relationship between median endoscopic healing index (EHI) and serum drug concentration quartiles in adult patients with Crohn’s disease treated with infliximab (A) or adalimumab (B). IFX: infliximab; ADA: adalimumab.


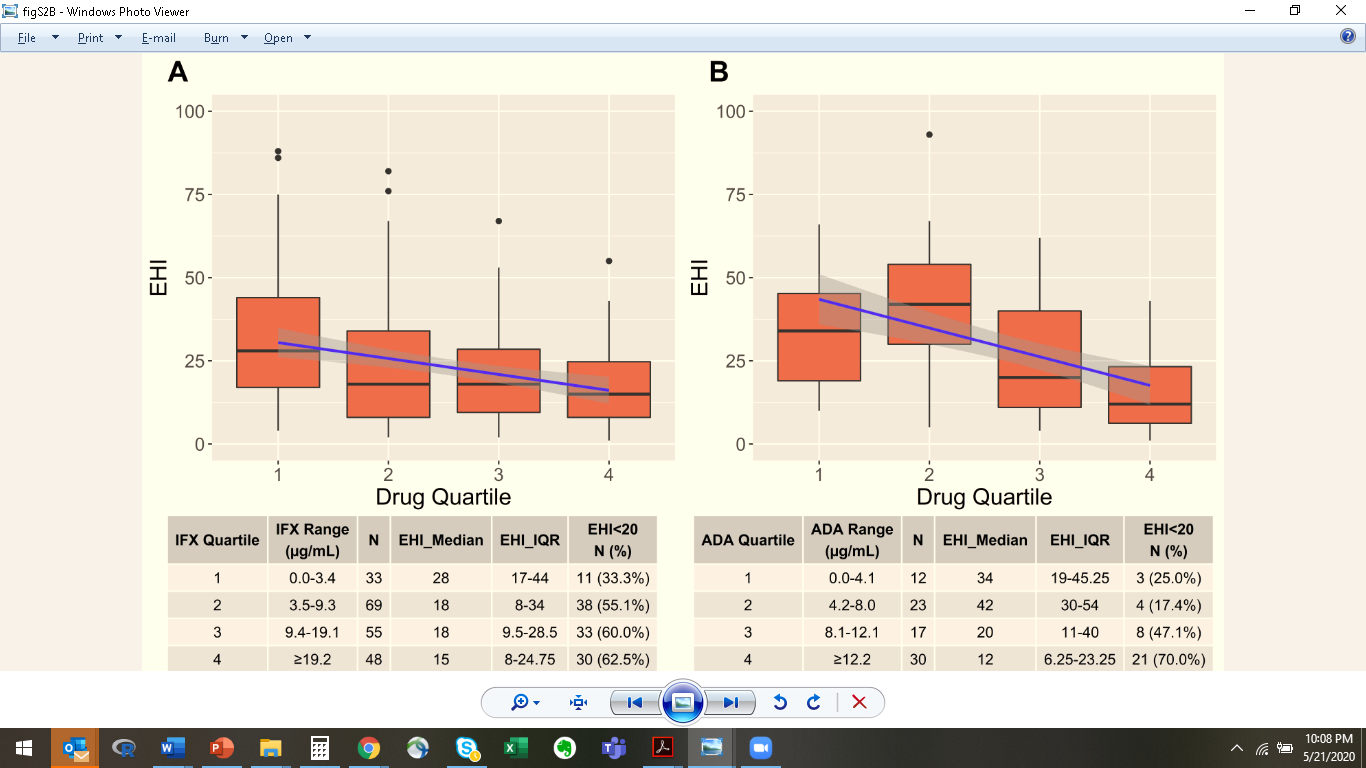


**Supplementary Figure 4.** Linear exposure-response relationship between median endoscopic healing index (EHI) and serum drug concentration quartiles in pediatric patients with Crohn’s disease treated with infliximab (A) or adalimumab (B). IFX: infliximab; ADA: adalimumab.
